# Estimates of cancer incidence and mortality in China, 2013

**DOI:** 10.1186/s40880-017-0234-3

**Published:** 2017-08-17

**Authors:** Rongshou Zheng, Hongmei Zeng, Siwei Zhang, Wanqing Chen

**Affiliations:** National Central Cancer Registry, National Cancer Center/Cancer Hospital, Chinese Academy of Medical Sciences and Peking Union Medical College, Beijing, 100021 P. R. China

**Keywords:** Incidence, Mortality, Cancer registry, Epidemiology, China

## Abstract

**Introduction:**

Population-based cancer registration data are collected by the National Central Cancer Registry in China every year. Cancer incident cases and cancer deaths in 2013 were analyzed.

**Methods:**

Through the procedure of quality control, reported data from 255 registries were accepted to establish the national database for cancer estimates. Incidences and mortalities were calculated with stratification by area (urban/rural), sex (male/female), age group (0, 1–4, 5–9, 10–14 … 80–84, and 85-year-old and above), and cancer site. The structure of Segi’s population was used for the calculation of age-standardized rates (ASR). Top 10 most common cancers and leading causes of cancer deaths were listed.

**Results:**

In 2013, 3,682,200 new cancer cases and 2,229,300 cancer deaths were estimated in China based on the pooled data from 255 cancer registries, covering 16.65% of the national population. The incidence was 270.59/100,000, with an ASR of 186.15/100,000; the mortality was 166.83/100,000, with an ASR of 108.94/100,000. The top 10 most common cancer sites were the lung, stomach, liver, colorectum, female breast, esophagus, thyroid, cervix, brain, and pancreas. The ten leading causes of cancer deaths were lung cancer, liver cancer, gastric cancer, esophageal cancer, colorectal cancer, pancreatic cancer, female breast cancer, brain tumor, leukemia, and lymphoma.

**Conclusions:**

Cancer leaves serious disease burden in China with high incidence and mortality. Lung cancer was the most common cancer and the leading cause of cancer death in China. Efficient control strategy is needed, especially for major cancers.

The National Central Cancer Registry (NCCR) collects data from local registries in China every year and publishes annual cancer statistics of China. In 2016, the collection of cancer registration data in 2013 was completed, and data analysis was done for publication. We reported the main results of cancer incidence and mortality in *China Cancer* [1] and *Cancer Letters* [2]. In the present paper, the cancer statistics were briefly introduced based on the published papers.

A total of 347 cancer registries submitted registration data of 2013 to NCCR in 2016. The number of registries increased compared with 261 cancer registries in 2012 [3]. Submitted data were checked following the quality control procedure. After several rounds of feedback and re-submission, the data from 255 registries were qualified for final database input and accepted for analysis. In local registries, data resources include hospitals, clinics, community health centers, medical insurance departments, and death registries. Demographic data were obtained from local statistical bureaus or household register departments in local public security bureaus. Proportion of morphological verification (MV %), percentage of cancer cases identified with death certification only (DCO %), mortality to incidence (M/I) ratio, percentage of uncertified cancer, and percentage of cancer with undefined or unknown primary site were the key indicators for evaluating data completeness, validity, and reliability. Changes of incidences and mortalities by year were also calculated. Pooled data were stratified by area (urban/rural), sex (male/female), age group (0, 1–4, 5–9, 10–14 … 80–84 85-year-old and above), and cancer site. The Chinese standard population in 2000 and the world Segi’s population were used for the calculation of age-standardized incidence/mortality rates. The nation-wide cancer incident cases and cancer deaths were estimated using age-specific rates stratified by area, sex, caner site, and corresponding national population in 2013.

The valid 255 cancer registries were composed of 88 urban and 167 rural registries, covering 226,494,490 citizens, accounting for 16.65% of the national population in 2013. The MV %, DCO %, and M/I ratio of overall cancers were 68.04, 1.74%, and 0.62, respectively.

We estimated that about 3,682,000 new cancer cases were diagnosed in 2013 (Table 1). The incidence of all cancers was 270.59/100,000 (293.79/100,000 in males and 246.21/100,000 in females). The age-standardized incidence rates adjusted by the Chinese standard population (ASIRC) and by the world Segi’s population (ASIRW) were 190.17/100,000 and 186.15/100,000. The cumulative incidence rate from birth to 74-year-old was 21.60%. The cancer incidence in urban areas was higher than that in rural areas (crude incidence 283.79/100,000 vs. 255.27/100,000; ASIRC 193.53/100,000 vs. 185.42/100,000; ASIRW 188.87/100,000 vs. 182.35/100,000).

**Table 1:** Estimated cancer incidence in China in 2013. This Table was published previously and is reproduced with permission from *China Cancer* [1]

Areas	Sex	Cases (×1000)	Crude incidence (1/10^5^)	ASIRC (1/10^5^)	ASIRW (1/10^5^)	Cumulative rate 0–74 years (%)
All	Both	3682.0	270.59	190.17	186.15	21.60
	Male	2048.6	293.79	212.30	210.74	25.00
	Female	1633.4	246.21	170.38	163.93	18.28
Urban	Both	2074.9	283.79	193.53	188.87	21.68
	Male	1117.6	299.49	209.81	207.91	24.48
	Female	957.3	267.43	179.95	172.54	19.06
Rural	Both	1607.1	255.27	185.42	182.35	21.45
	Male	931.0	287.24	215.07	213.95	25.62
	Female	676.1	221.34	157.71	152.73	17.25

*ASIRC* age-standardized incidence rate adjusted by the China standard population in 2000, *ASIRW* age-standardized incidence rate adjusted by the world Segi’s population

About 2,229,300 cancer deaths were estimated in 2013 all over the country (Table 2). The crude mortality was 163.83/100,000 (201.67/100,000 in males and 124.06/100,000 in females), and age-standardized mortality rates adjusted by the Chinese standard population (ASMRC) and by the world Segi’s population (ASMRW) were 109.95/100,000 and 108.94/100,000. The cumulative mortality rate from 0 to 74-year-old was 12.33%. The cancer mortality in urban areas were lower than those in rural areas (crude mortality 161.48/100,000 vs. 166.57/100,000; ASMRC 104.57/100,000 vs. 116.42/100,000; ASMRW 103.65/100,000 vs. 115.23/100,000).

**Table 2:** Estimated cancer mortality in China in 2013. This Table was published previously and is reproduced with permission from China Cancer [1]

Area	Sex	Deaths (×1000)	Crude mortality (1/10^5^)	ASMRC (1/10^5^)	ASMRW (1/10^5^)	Cumulative rate 0–74 years (%)
All	Both	2229.3	163.83	109.95	108.94	12.33
	Male	1406.2	201.67	143.08	142.39	16.22
	Female	823.1	124.06	78.57	77.37	8.46
Urban	Both	1180.6	161.48	104.57	103.65	11.44
	Male	735.7	197.16	134.80	134.33	15.01
	Female	444.9	124.28	76.05	74.80	7.95
Rural	Both	1048.7	166.57	116.42	115.23	13.38
	Male	670.5	206.86	152.93	151.87	17.65
	Female	378.2	123.81	81.59	80.43	9.06

*ASMRC* age-standardized mortality rate adjusted by the China standard population in 2000, *ASMRW* age-standardized mortality rate adjusted by the world Segi’s population

For males, lung cancer was still the most common cancer, with 488,800 new cases, accounting for nearly 1/4 of all cancers (Fig. 1). Gastric cancer ranked the second, followed by liver cancer, colorectal cancer, esophageal cancer, prostate cancer, bladder cancer, pancreatic cancer, lymphoma, and central nerve system tumors. Prostate cancer, which ranked the seventh in 2012 [3], rose to the sixth place in male cancer spectrum. Incidences of colorectal, thyroid, prostate, and pancreatic cancers were higher in urban areas than in rural areas, whereas gastric, liver, and esophageal cancers were more common in rural areas (Fig. 2). Lung cancer ranked the top both in urban and rural areas with similar incidences.

**Fig. 1:**
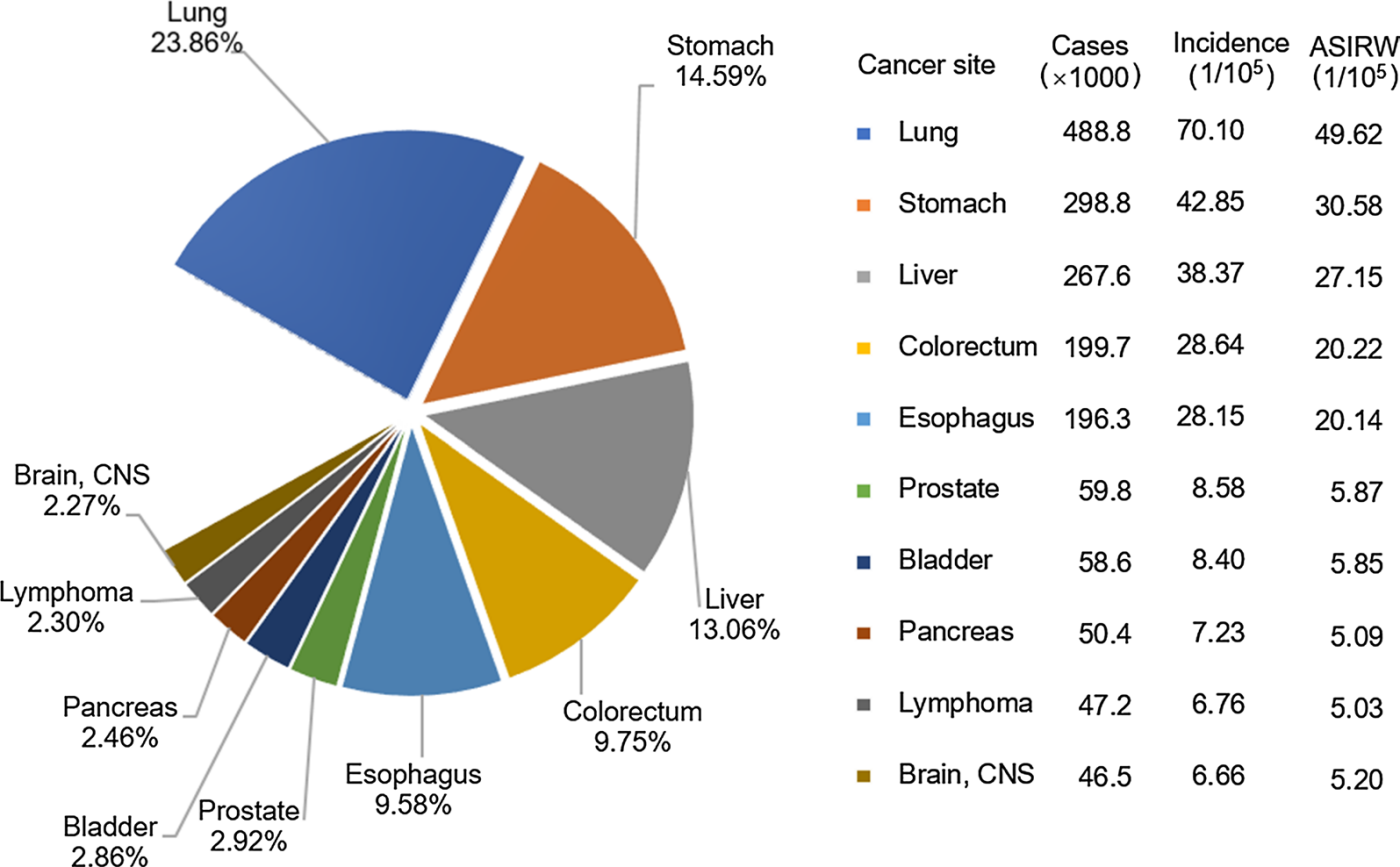
Incidences of top 10 cancers for males in China, 2013. *ASIRW* age-standardized incidence rate adjusted by the world Segi’s population, *CNS* the central nervous system

**Fig. 2:**
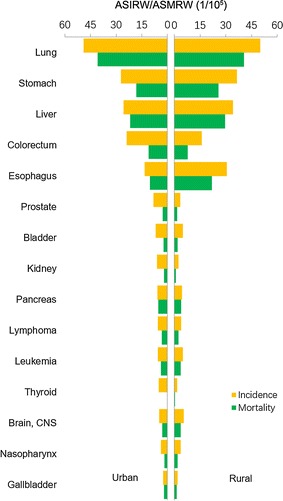
ASIRW and ASMRW in urban areas and rural areas for males in China, 2013. *ASMRW* age-standardized mortality rate adjusted by the world Segi’s population

For females, breast cancer is the most common cancer, with 278,800 new cases (Fig. 3). The incidence of thyroid cancer kept increasing, and thyroid cancer became the fifth most frequent cancer. Incidences of breast, lung, colorectal, thyroid, ovarian cancers were higher in urban areas than in rural areas, whereas gastric, liver, and esophageal cancers were more common in rural areas (Fig. 4).

**Fig. 3:**
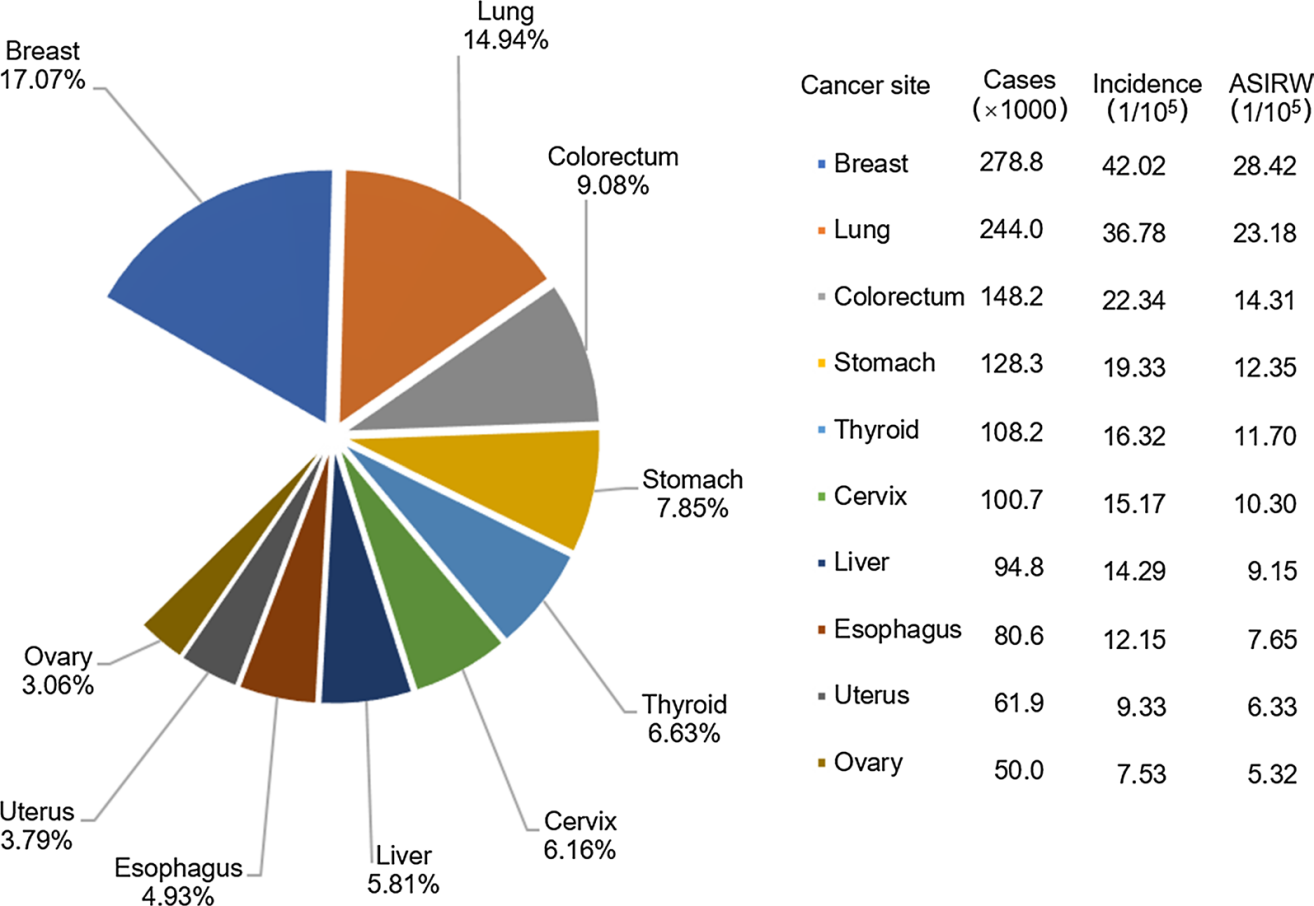
Incidences of top 10 cancers for females in China, 2013

**Fig. 4:**
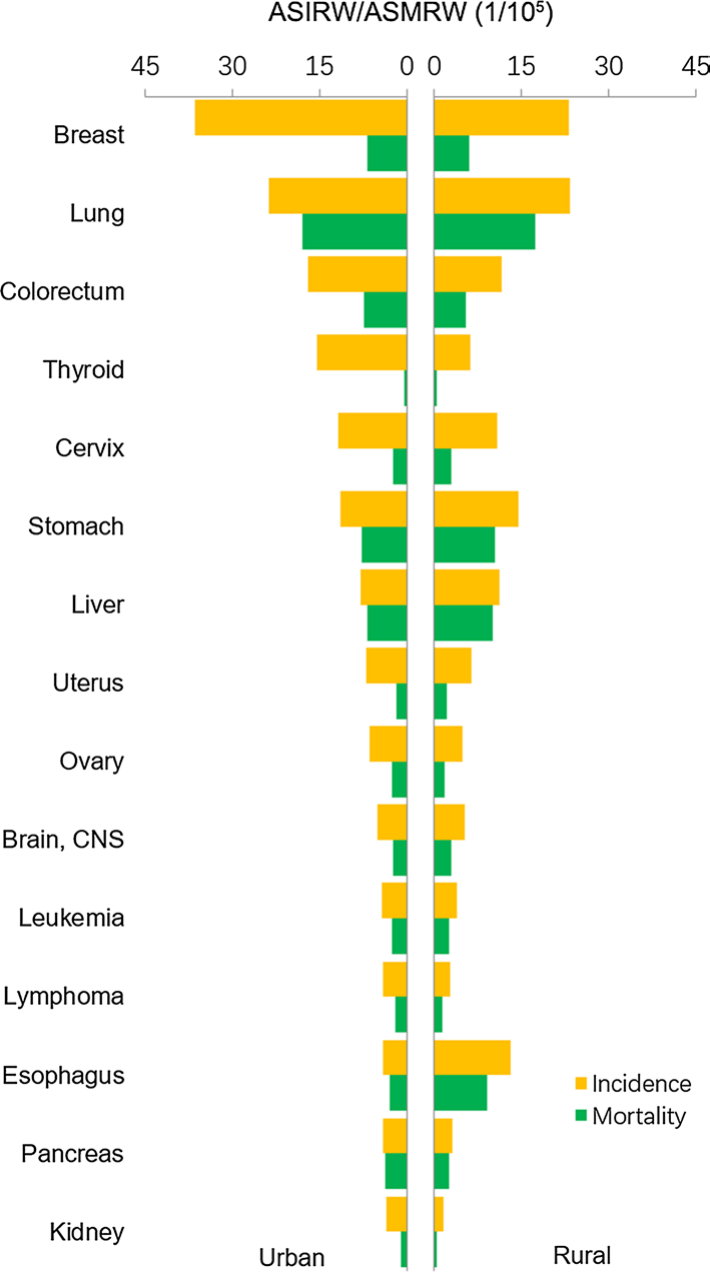
ASIRW and ASMRW in urban areas and rural areas for females in China, 2013

Lung cancer was the leading cause of cancer death in China both in males and females in 2013 (Figs. 5, 6). Liver cancer ranked the second, followed by gastric, esophageal, and colorectal cancers in males (Fig. 5). For females, cancers of gastric, liver, colorectum, and breast ranked the second to fifth most fetal causes of cancer death (Fig. 6). Mortalities of lung, liver, gastric, and esophageal cancers were higher in males than in females. For both sexes, although mortalities of major cancers were different in urban and rural areas, the cancer death patterns were almost the same (Figs. 2, 4). Significant differences with pervious statistics were that prostate cancer incidence ranked the sixth for males in urban areas and colorectal cancer became the second common cause of cancer deaths for females in urban areas in 2013, which ranked the seventh and third in 2012 [3].

**Fig. 5:**
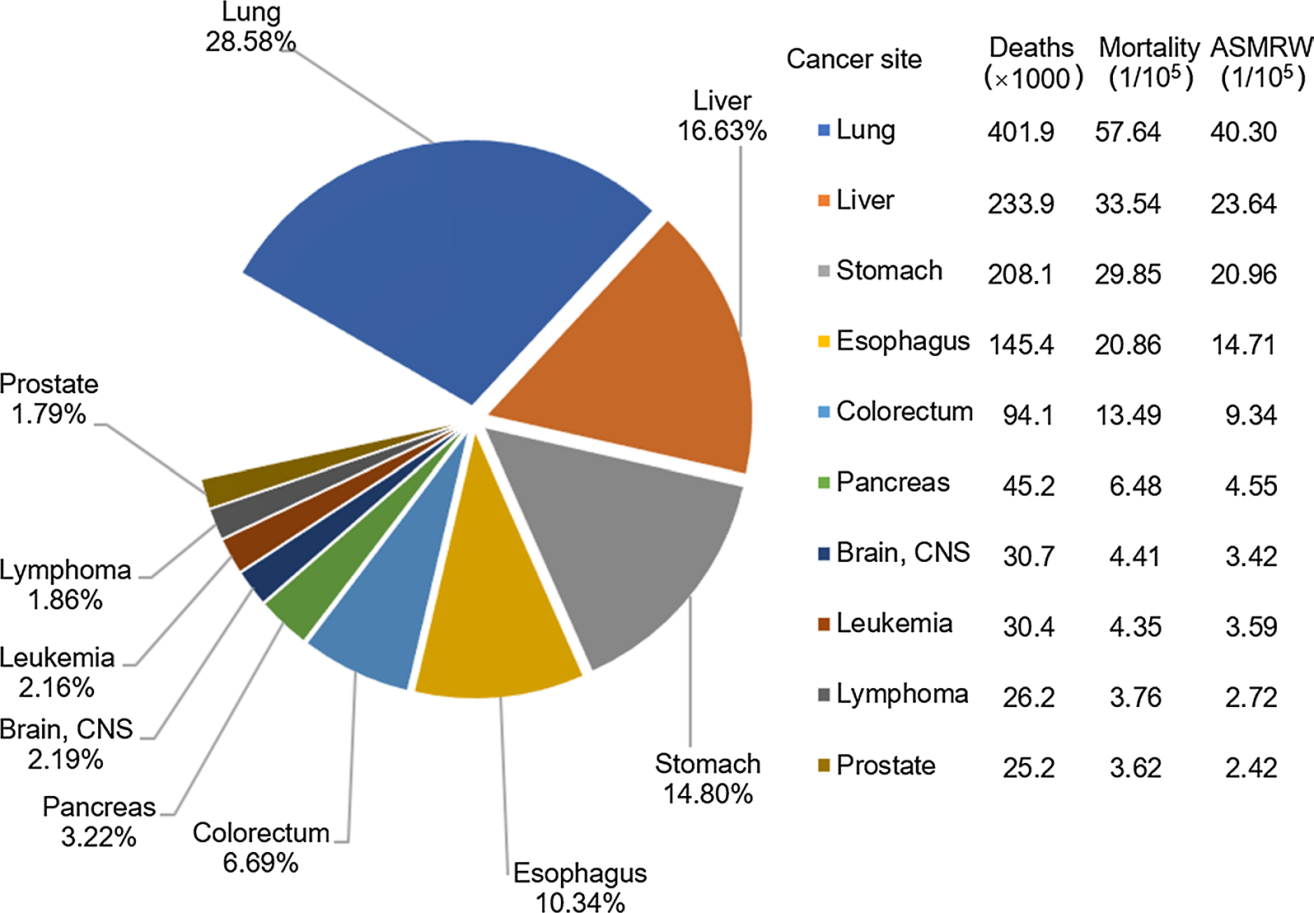
Mortalities of top 10 cancers for males in China, 2013

**Fig. 6:**
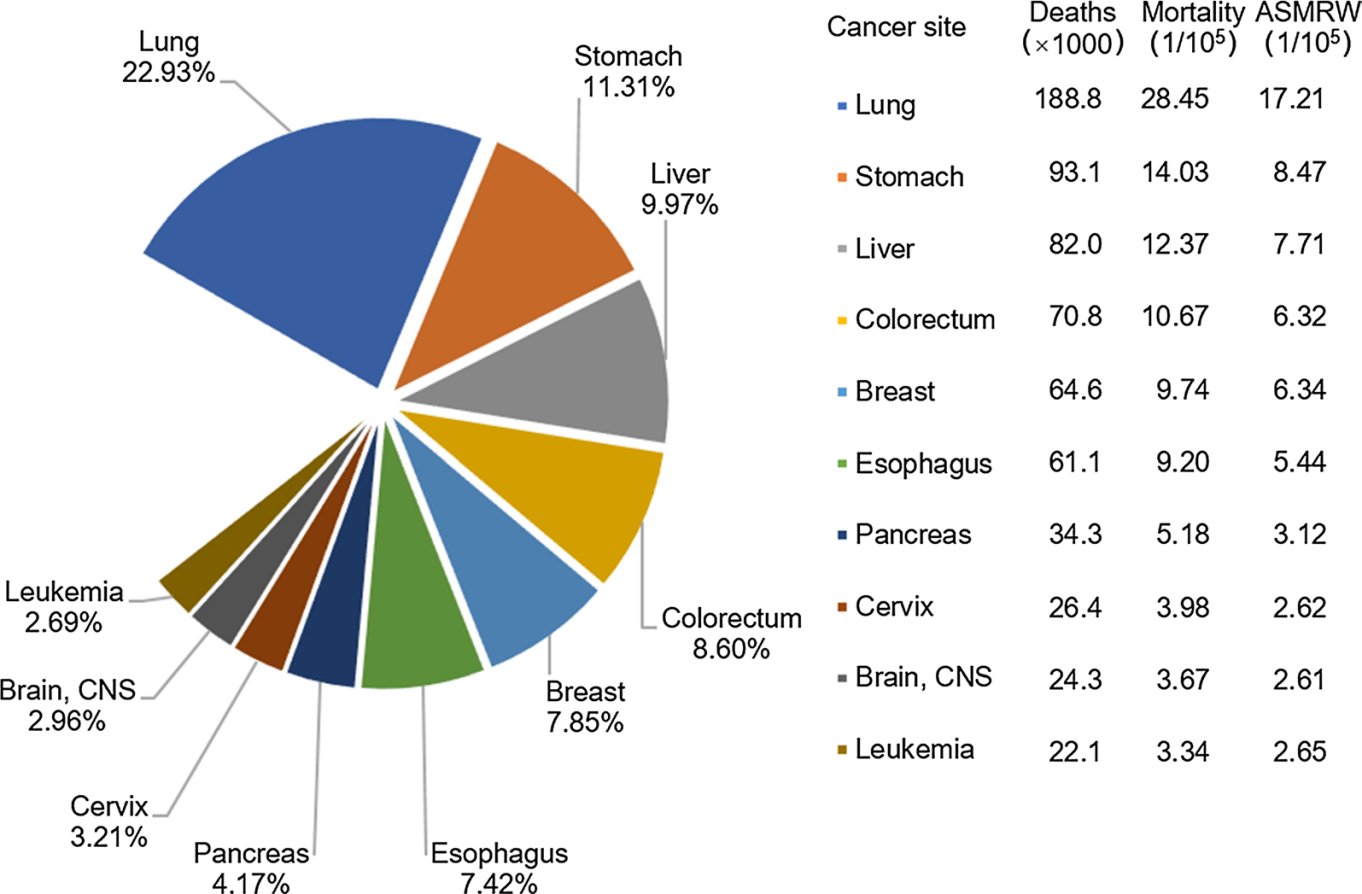
Mortalities of top 10 cancers for females in China, 2013

The cancer burden still increased compared with that in 2012 [4], even though cancer incidences and mortalities did not change markedly. The coverage of cancer registry keep rising and data quality improving. The estimates of cancer new cases and cancer deaths get more accurate and reliable. As the basis of making anti-cancer policy, conducting research, and providing health care, cancer registration plays a key role in cancer prevention and control in China.

## References

[1] Chen W, Zheng R, Zhang S, Zeng H, Zou X, He J (2017). Report of cancer incidence and mortality in China, 2013. China Cancer.

[2] Chen W, Zheng R, Zhang S, Zeng H, Xia C, Zuo T (2017). Cancer incidence and mortality in China, 2013. Cancer Lett.

[3] Chen W, Zheng R, Zeng H, Zhang S (2016). The incidence and mortality of major cancers in China, 2012. Chin J Cancer.

[4] Chen W, Zheng R, Zuo T, Zeng H, Zhang S, He J (2016). National cancer incidence and mortality in China, 2012. Chin J Cancer Res.

